# Bibliographical

**Published:** 1896-04

**Authors:** 


### Bibliographical.

Catching's Compendium of Practical Dentistry for 1895. By B. H. Catching, D.D.S., editor and publisher, Atlanta, Ga.

This very valuable work for 1895 has just come to hand, and is quite an improvement over the former editions. The subjects are so arranged and systematized as to be easily found. The work is one of great value, as its aim has been, and this has been quite fully accomplished by placing here in accessible form the very pith and marrow of the journalistic literature of this and other countries. While, perhaps, the leading object has been to give matters pertaining more particularly to the practical side of our profession, yet its principles and science have not been overlooked. In the production of this work Dr. Catching has performed a great service for the busy practitioner, and not only this, but it will be a work of ready reference for the college student and, may we say it, for the professor as well. Dr. Catching is entitled to the thanks of the entire dental fraternity for the accomplishment of this work. It should be in the hands of every dentist and every dental student in this country, and also in other countries, especially where the English language is spoken. As Dr. Catching goes on from year to year in this work he becomes better and better fitted for it; learns more and more just what is adapted to the needs of the profession, and, although it is difficult to conceive how improvements could be made on this edition of 1895, yet there is no question but that Dr. Catching, having the subject constantly in mind, will, from year to year, make further improvements.

